# Impact of exercise intervention on immune function during aging

**DOI:** 10.3389/fimmu.2026.1817225

**Published:** 2026-07-29

**Authors:** Junyu Bai, Jinqiao Zhang

**Affiliations:** School of Physical Education, Shaanxi Normal University, Xi’an, China

**Keywords:** aging, cellular senescence, exercise intervention, immunosenescence, inflammation

## Abstract

Global population aging faces immunosenescence, increasing infection risk, reducing vaccine efficacy, and raising chronic disease burden, a major public health challenge. Identifying safe, effective interventions is paramount. This review evaluates the potential of regular exercise as a non-pharmacological strategy to counteract key features of immunosenescence. Exercise acutely redistributes immune cells and, via long-term adaptation, triggers coordinated effects including neuroendocrine modulation, myokine release, metabolic remodeling, and gut microbiota improvement. These adaptations appear to reduce chronic inflammation, may help optimize adaptive immune cell repertoires, and are associated with enhanced immune surveillance. Exercise type, intensity, and duration uniquely influence immunomodulatory outcomes, underscoring the need for personalized prescriptions. Current research supports the immunoprotective benefits of exercise; however, gaps remain regarding the functional significance of exercise-induced biomarker changes, the differential activation of mechanistic pathways across exercise modalities, and the translation of short-term biomarker improvements into reduced infection risk or enhanced vaccine responses in older adults. Future studies must address these issues to advance evidence-based, precise exercise recommendations for healthy aging.

## 1 Introduction

Age-related decline in immune function poses a major public health challenge for aging populations. The concept of immunosenescence was first introduced in the late 1970s to 1980s to describe the age-related functional decline of the adaptive immune system, particularly T-cell-mediated immunity (1, 2). By the 1990s and 2000s, the definition had expanded into an umbrella term encompassing impaired innate immunity, chronic low-grade inflammation (later termed inflammaging), and classical cellular markers such as loss of CD28 expression, telomere shortening, and restricted T-cell receptor repertoire diversity (3–5). This expansion, however, introduced a caveat: it conflated age-associated immune remodeling with genuine aging damage. In the past decade, a more nuanced framework has emerged, positing that many changes traditionally classified as immunosenescence—including CD28 loss and oligoclonal memory T-cell expansion—may represent adaptive compensatory responses to lifelong antigenic pressure, rather than passive decay (6, 7). Furthermore, no consensus biomarker panel exists to define an “immunosenescent” individual, and the predictive value of these markers for clinical endpoints (e.g., infection susceptibility, vaccine response, and frailty) remains inconsistent (8–10). In essence, the definition has shifted from a linear decline model, through an expanded umbrella concept, to a heterogeneous remodeling framework that distinguishes adaptation from damage.

The term inflammaging was coined by Franceschi and colleagues in 2000 to describe the chronic, low-grade, sterile systemic inflammation that progressively intensifies with age (1). Over the ensuing two decades, inflammaging has been recognized as a hallmark of aging and a mechanistic link to multiple age-related diseases (2, 5). Despite broad acceptance, key ambiguities persist: “low-grade” inflammation lacks an agreed quantitative threshold; inflammaging and immunosenescence are intertwined and mutually reinforcing; and emerging evidence suggests that a moderate age-related inflammatory tone may even serve beneficial adaptive functions (11–15) Therefore, any anti-inflammatory strategy targeting immunosenescence—including exercise—should aim to normalize, rather than eliminate, systemic inflammation, and studies must distinguish the reduction of pathological inflammation from the blunting of potentially adaptive immune responses (4, 16, 17). These critical considerations are essential for evaluating exercise as an intervention against immunosenescence. Regular physical activity, a safe and accessible lifestyle intervention, is increasingly recognized for its immunomodulatory effects (18, 19).

Exercise influences immunity through multiple mechanisms: at the physiological level, it reprograms immune cell metabolism and function (20); at the tissue level, skeletal muscle acts as an endocrine organ via resident immune cells (21–23); clinically, it preserves immune competence and mental well-being (24–26) while improving cancer survivors’ quality of life (27, 28); at the molecular level, exercise shapes immunity through epigenetic pathways and stress response modulation (29, 30).

Accumulating evidence suggests that regular exercise attenuates immunosenescence by targeting immune cell metabolism, epigenetic remodeling, and neuroendocrine signaling. However, the following issues remain to be further clarified: (i) the respective characteristics of the acute and chronic effects of exercise on immunosenescence biomarkers, and their actual implications for immune function; (ii) how the major mechanistic pathways of exercise-induced immune regulation—including myokine signaling, neuroendocrine modulation, autophagy, and gut microbiota remodeling—are differentially activated by different exercise modalities; and (iii) whether short-term improvements in biomarkers can translate into reduced infection risk or enhanced vaccine responses in diverse older populations. This review addresses these issues by synthesizing current evidence and proposing a framework for personalized exercise prescription.

This narrative review synthesized literature on exercise and immune aging. We searched PubMed, Web of Science, and Scopus up to February 2026 using terms related to exercise, immune function, and aging. Key original and review articles were prioritized to provide a comprehensive overview. Reference lists were also screened for additional relevant studies.

Throughout this review, we distinguish between direct evidence obtained from older adult populations and mechanistic insights derived from studies in younger adults or animal models that may inform our understanding of aging. Where direct evidence in older adults is limited, we explicitly note this and frame the findings as promising directions requiring further validation in aging cohorts.

## 2 Key characteristics and health consequences of immunosenescence

**Figure 1:**
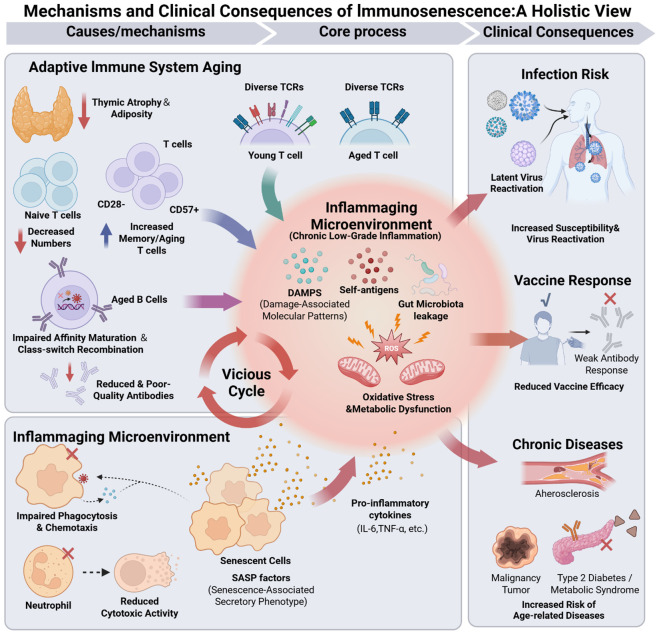
Immunosenescence: mechanisms and clinical consequences. Arrows indicate directional flow of processes and observed associations between biological events. This schematic illustrates three interconnected phases of immunosenescence: age-related adaptive and innate immune alterations as initiating factors, self-amplifying inflammaging as the central process, and its associated downstream clinical outcomes including heightened infection susceptibility, reduced vaccine efficacy and elevated age-related chronic disease risk. The left panel depicts adaptive immune aging initiated by thymic involution and adiposity (31–33), leading to reduced peripheral naive T cell output, accumulation of terminally differentiated CD28^-^CD57^+^ memory T cells (31–33), and decline in T cell receptor (TCR) repertoire diversity that limits responses to novel antigens (34). B cell dysfunction is manifested as impaired antibody affinity maturation and class-switch recombination, weakening humoral immunity (35–39). The lower left panel shows innate immune aging characterized by decreased phagocytic, chemotactic and cytotoxic activities in macrophages and neutrophils as well as reduced NK cell function (40–46). The central panel depicts inflammaging arising from accumulation of senescent cells secreting SASP factors (47–49), persistent low-level immune responses to endogenous stimuli (self-antigens, DAMPs, microbiota), and age-related metabolic alterations with oxidative stress (11, 50–58), systemically elevating circulating pro-inflammatory cytokines and acute-phase proteins (59–61). The right panel shows clinical consequences: increased infection susceptibility and latent virus reactivation (65, 66), attenuated vaccine immunogenicity from diminished naive T cells and impaired B cell function (36, 67–69), and elevated chronic disease risk including atherosclerosis, type 2 diabetes and malignancy (70, 71). Created in BioRender. https://BioRender.com/yqu8cic (License: WR29THB30W).

Immunosenescence drives age-related immune deterioration through four interconnected domains (**Table 1**): adaptive immune remodeling, innate immune dysregulation, establishment of inflammaging, and the consequent clinical sequelae. This framework integrates the cellular, molecular, and clinical dimensions of immune aging (**Figure 1**).

**Table 1:** Summary of the multi-level features and clinical consequences of immunosenescence.

Aspect of immunosenescence	Core features	Cellular/molecular mechanisms	Functional consequences	References
Adaptive Immune System Changes	• Thymic involution & ↓ output • ↓ naïve T cells • Accumulation of CD28^+^CD57^+^ senescent T cells • ↓ TCR repertoire diversity • Impaired B cell affinity maturation & class switching	• Thymic involution • Age-related lymphocyte alterations	• Impaired response to new antigens • ↓ humoral immunity • Reliance on memory responses • ↑ vulnerability to new antigens	(31–39)
Innate Immune System Changes	• ↓ phagocytic, chemotactic, cytotoxic activities • ↓ NK cell function • SASP release from senescent immune cells • Chronic inflammation → further innate suppression	• Functional decline of innate cells • SASP secretion • Self-perpetuating cycle	• Impaired pathogen defense • Mutual reinforcement of immune dysfunction and persistent inflammation	(40–49)
Inflammaging	• Chronic low-grade inflammation • ↑ pro-inflammatory cytokines & acute-phase proteins	• Senescent cell SASP • Persistent responses to endogenous stimuli • Age-related metabolic alterations + oxidative stress	• Links immunosenescence to age-related diseases • Convergence point for aging alterations • Perpetuates pathogenic vicious cycle	(11, 50–60)
Clinical Implications	• ↓ host defense, homeostatic failure • ↑ susceptibility to infections & latent virus reactivation • Attenuated vaccine immunogenicity • ↑ chronic disease risk	• ↓ naïve T cells • Impaired B cell function • Inflammaging drives atherosclerosis & T2D • Waning immune surveillance → malignancy	• Poor vaccine responses • Links aging to geriatric syndromes • Targeting immunosenescence may reduce age-related diseases & preserve health	(36, 61–71)

CVD, cardiovascular disease; DAMP, damage-associated molecular pattern; SASP, senescence-associated secretory phenotype; TCR, T cell receptor; T2D, type 2 diabetes. Arrows (↑/↓) indicate increase or decrease, respectively. Reference numbers correspond to the original sources as cited in the main text.

As people age, immunosenescence is marked by a widespread transformation of the adaptive immune system. The starting point is the shrinking of the thymus and decreased thymic production, resulting in fewer naïve T cells in the periphery and a buildup of terminally differentiated memory and aging (e.g., CD28^-^CD57^+^) T cells (31–33). The reduction in T cell receptor (TCR) repertoire diversity limits the ability to respond to new antigens (34). B cell function is also compromised, with impaired antibody affinity maturation and class-switch recombination weakening humoral immunity (35–39). These age-related alterations constitute the cellular foundation of immunosenescence.

Alongside these changes, the innate immune system also undergoes a functional decline, characterized by decreased phagocytic, chemotactic, and cytotoxic activities in macrophages and neutrophils, as well as reduced NK cells function (40–46). These impairments weaken pathogen defense. Senescent immune cells release senescence-associated secretory phenotype (SASP) factors, driving chronic inflammation that further suppresses innate immunity, creating a self-perpetuating cycle (47–49). Thus, innate immunosenescence encompasses both functional decline and dysregulation, fostering a self-perpetuating cycle of immune dysfunction and persistent inflammation, termed inflammaging.

“Inflammaging” arises from multifactorial mechanisms: accumulation of senescent cells secreting SASP factors, persistent low-level immune responses to endogenous stimuli (self-antigens, DAMPs, microbiota), and age-related metabolic alterations with oxidative stress (11, 50–58). Systemically, it elevates circulating pro-inflammatory cytokines and acute-phase proteins (59–61). Beyond being a sign of immune dysfunction, inflammaging acts as an essential pathophysiological link between immunosenescence and age-associated conditions such as cardiovascular and neurodegenerative disorders (62). It serves as a convergent point for diverse aging-related alterations while perpetuating a vicious cycle that drives pathogenesis (63, 64).

Immunosenescence clinically manifests as declining host defense and homeostatic failure. This includes increased susceptibility to infections and latent virus reactivation (65, 66); attenuated vaccine immunogenicity from diminished naïve T cells and impaired B cell function, leading to poor antibody responses to influenza, pneumococcal, and SARS-CoV-2 vaccines (36, 67–69); and elevated chronic disease risk, where inflammaging drives atherosclerosis and type 2 diabetes while waning immune surveillance permits malignancy (70, 71). Collectively, these immune−mediated processes form a mechanistic nexus linking biological aging to geriatric syndromes and age−related multimorbidity.

## 3 Modulatory effects of exercise intervention on innate immune function

**Figure 2:**
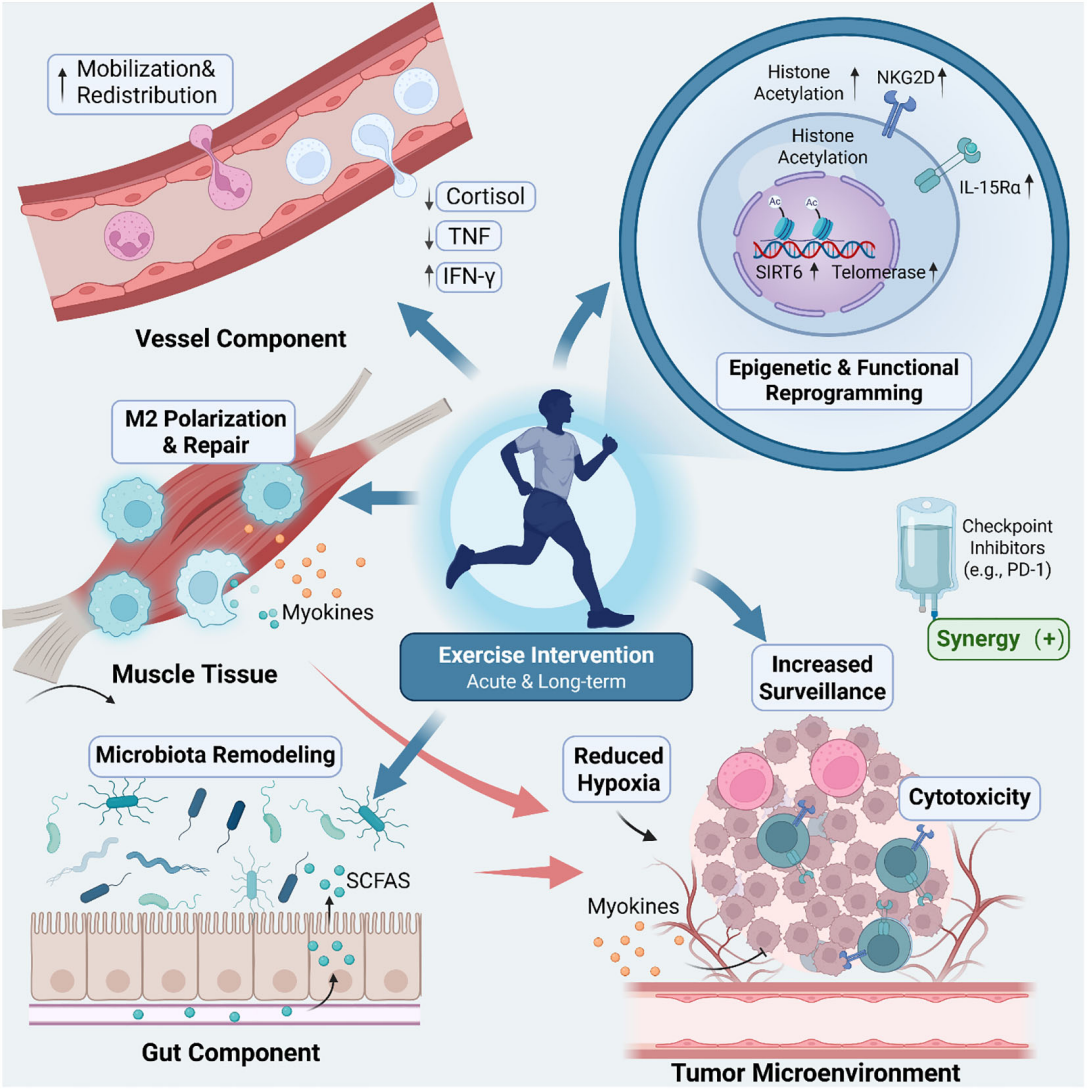
Multi-level mechanisms of exercise-induced immune modulation. Arrows denote pathways or mechanistic links supported by experimental evidence. Upward arrows (↑) denote increased expression or activity; downward arrows (↓) denote decreased expression or activity. This schematic illustrates that acute and long-term exercise intervention exerts antitumor effects through systemic pathways: mobilizing immune cells and regulating cytokine release via vascular circulation (72); secreting myokines from skeletal muscle, with IL-6 released abundantly during exercise exerting anti-inflammatory effects by stimulating IL-10 and inhibiting TNF-α (73–75), and IL-15 promoting NK cell development and CD8^+^ T cell function (76–80); remodeling gut microbiota and increasing SCFA production (81, 82). These changes collectively drive immune cell epigenetic and functional reprogramming via histone modifications (83–85), reduce tumor hypoxia, enhance intratumoral immune surveillance and cytotoxicity through CD8^+^ T cell infiltration via CXCR3 signaling (86–88), and show synergistic effects with immune checkpoint inhibitors (89, 90). Endurance exercise increases reparative M2-like macrophages in muscle (91, 92). Post-exercise serum enhances NK cell cytotoxicity, associated with reduced cortisol and elevated IFN-γ (93). Acute exercise mobilizes effector and memory T cells via catecholamine-mediated redistribution (94, 95) and elevates histone acetylation with NKG2D upregulation (96). The tumor microenvironment data derive almost exclusively from syngeneic mouse tumor models (86–88, 97–99), and the hypothesis that exercise synergizes with immune checkpoint inhibitors has not been tested in randomized cancer patient trials (89, 90). Created in BioRender. https://BioRender.com/u24dk8m (License: IA29THAQGQ).

Exercise modulates innate immune function through multiple interrelated mechanisms (**Figure 2**). These include phase-specific effects on neutrophils and monocytes/macrophages, multi-level regulation of NK cell activity, dose-dependent modulation of systemic inflammatory balance, and enhancement of immune surveillance with implications for anti−tumor immunity.

### 3.1 Effects of acute and long-term exercise on neutrophil and monocyte/macrophage function

Evidence from human intervention studies and meta-analyses indicates that exercise modulates neutrophil and monocyte/macrophage function through multiple mechanisms. Acute exercise rapidly alters monocyte metabolic state and suppresses pro-inflammatory cytokine production via β2-adrenergic activation, a catecholamine-driven process detailed in Section 5.1 (20, 72). It modifies neutrophil gene expression, influences apoptosis through oxidative stress, and promotes recruitment to skeletal muscle (100–102). Human studies show that endurance exercise increases reparative M2-like macrophages in muscle, while chronic exercise modulates macrophage polarization in metabolic tissues of aged mice, reducing inflammation (91, 92). Thus, acute exercise triggers rapid mobilization and regulation, whereas long-term training appears to shift the tissue immune microenvironment toward a pro-homeostatic and reparative phenotype, though the available evidence on these chronic adaptations remains largely indirect.

### 3.2 Exercise’s influence on natural killer cell numbers and their cytotoxic function

Exercise influences NK cells through both acute mobilization and potential long−term functional enhancement. In physically active adults, moderate aerobic training has been associated with increased NK cell cytotoxicity (93). Whether these effects are preserved in older adults remains to be fully established. Human studies across a range of ages have shown that acute intense exercise transiently increases circulating NK numbers via catecholamine-mediated redistribution (see Section 5.1 for neuroendocrine mechanisms) (94, 95). Intense endurance exercise elevates histone acetylation and upregulates NKG2D (96). Post-exercise serum enhances cytotoxicity, associated with reduced cortisol and elevated IFN-γ (93). Thus, exercise regulates NK cells through integrated cellular redistribution, epigenetic modifications, and humoral factor changes. Beyond regulating specific cell types, exercise exerts broader effects on systemic inflammatory balance.

### 3.3 Exercise and inflammatory balance

A single bout of exercise triggers a transient, cytokine-mediated inflammatory-like response that resolves during recovery. Acute exercise induces a marked but short-lived elevation of circulating pro-inflammatory cytokines such as IL-6 and TNF-α, which return to baseline within hours of exercise cessation (103). In contrast, regular long-term exercise exerts a net anti-inflammatory effect. Regular moderate-intensity exercise helps reduce the persistent low-level inflammation associated with aging and various chronic diseases (104, 105). The anti-inflammatory impact functions via several pathways: directly altering immune cell functions and indirectly benefiting from better metabolic and cardiovascular health (106–110). Additional anti-inflammatory mechanisms, including myokine-mediated cytokine modulation and gut microbiota remodeling, are discussed in Section 5. Thus, regular exercise appears to shift systemic inflammatory balance toward a net anti-inflammatory state through multiple complementary pathways, though whether this shift is sustained after exercise cessation remains unclear. By relieving the chronic inflammatory burden that characterizes immunosenescence, this anti-inflammatory milieu may represent a key mechanism through which exercise counters immune aging.

### 3.4 Exercise-induced enhancement of immune surveillance and its potential role in anti-tumor immunity

Emerging evidence, primarily from preclinical studies, suggests that exercise may enhance immune surveillance and anti-tumor immunity through multi-layered mechanisms. Studies in humans and experimental models have shown that epigenetically, exercise modulates telomerase, SIRT6, miRNAs, and inflammation/aging genes via histone modifications, preserving homeostasis (83–85). Human and animal studies indicate that HIIT boosts CD8^+^ T cell cytotoxicity, while gut metabolites and IL-15/IL-15Rα signaling potentiate anti-tumor function (97–99). Studies in tumor-bearing mice have demonstrated that in the tumor microenvironment (TME), exercise alleviates hypoxia, promotes CD8^+^ infiltration via CXCR3, and suppresses stromal formation via myokines, creating an immune-permissive environment (86–88), a concept supported by mechanistic reviews (111). It has been proposed that macrophage polarization induced by exercise and reduced systemic inflammation may help prevent cancer in the elderly (112). Reviews of preclinical studies suggest that exercise may synergize with immune checkpoint inhibitors, representing a promising translational direction that warrants further investigation in clinical settings (89, 90). However, the current evidence linking exercise to enhanced anti-tumor immunity remains heavily weighted toward preclinical models. The tumor microenvironment data derive almost exclusively from syngeneic mouse tumor models, and their direct translatability to human biology requires validation. In humans, evidence is largely limited to observational studies showing inverse associations between physical activity and cancer incidence, rather than direct demonstration of enhanced tumor-infiltrating lymphocyte function or improved oncological outcomes (113). The hypothesis that exercise synergizes with immune checkpoint inhibitors has not been tested in randomized cancer patient trials. Furthermore, whether these anti-tumor immune effects are preserved in older adults—who harbor both immunosenescence and an elevated tumor mutational burden—remains unexplored. Collectively, these findings suggest that exercise may bolster immune surveillance and complement anti-cancer treatment; however, the evidence base remains predominantly preclinical, and direct demonstration of enhanced anti-tumor immune responses in exercising older adults is still lacking.

## 4 Modulatory effects of exercise intervention on adaptive immune function

### 4.1 Effects of physical activity on T−cell subset distribution

Exercise modulates T cell subsets in both the acute and chronic setting. Acutely, exercise mobilizes effector and memory T cells into circulation (see 4.2). Chronically, regular training reshapes the T cell repertoire, with effects dependent on modality and individual status. Randomized controlled trials and mechanistic studies have shown that long-term moderate aerobic training increases total lymphocytes and CD8^+^ effector memory (TEM) cells while reducing senescent CD8^+^ effector memory RA (EMRA) cells, with expanded TEM cells showing enhanced cytotoxicity via IL-15 (114–119). Conversely, a controlled trial in young men found that HIIT may reduce CD4^+^ T cells, naïve CD4^+^ T cells, and CD4:CD8 ratio (114, 115). Cross-sectional studies in older adults have shown that higher activity levels correlate with favorable naïve/memory ratios, suggesting sustained exercise preserves a youthful immune profile and TCR diversity, counteracting thymic involution-driven T cell aging (120, 121). Overall, current evidence suggests that regular exercise may help to maintain a balanced T cell profile and TCR diversity, potentially counteracting the decline in T cells associated with aging, though the long-term clinical significance of these changes remains to be established.

### 4.2 Exercise-induced enhancement of T cell proliferation, activation, and effector function

Physical activity has been shown to enhance the proliferation, activation, and function of T cells. Human studies have shown that acute exercise mobilizes effector and memory T cells into circulation and increases migration to peripheral tissues (122). Animal models and young human studies suggest that long-term training improves mitochondrial function in CD8^+^ T cells, including tumor-infiltrating lymphocytes, enhancing proliferative capacity and cytotoxicity (123–125). Translation of these mechanisms to the aging context requires further investigation. This process integrates neuroendocrine, metabolic, and vascular regulation (20, 125–127). A systematic review of human trials has shown that resistance training may modulate T cell phenotype and function in part via myokine release from skeletal muscle, as detailed in Section 5.2 (128–131). Whether these effects are fully recapitulated in older adults remains an active area of investigation. Observational studies have linked increased cardiorespiratory fitness to a smaller percentage of aging T cells, preserving a robust T cell repertoire (120). Studies in mouse models of breast cancer have demonstrated that exercise promotes CD8^+^ T cell infiltration and function through CXCR3 signaling, which provides a mechanistic rationale that is now being explored in preclinical and early-phase clinical studies (88). Thus, exercise appears to boost T cell effector function via synergistic pathways, though the majority of evidence derives from studies in younger adults or animal models, and direct confirmation of these effects in older populations remains limited. These enhancements in T cell function also have implications for B cell-mediated humoral immunity.

### 4.3 The impact of exercise on B cell function and antibody response

While direct evidence is limited, current findings indicate that regular exercise indirectly boosts humoral immunity by optimizing overall immune balance. As a non-pharmacological strategy to delay immunosenescence, exercise addresses the immunological underpinnings of blunted vaccine responses in older adults (7, 132). Observational studies and mechanistic reviews suggest that engaging in regular exercise reduces the accumulation of senescent T cells and mitigates chronic inflammation by enhancing immune cell metabolism and function, thereby establishing a systemic environment conducive to efficient and durable B−cell responses (120, 133–135). Furthermore, animal models have shown that exercise may also directly modulate B cell activity, as observed in certain models where it upregulates the inhibitory receptor FcγRIIB, exerting anti-inflammatory and immunomodulatory effects (136). Therefore, while direct evidence remains limited, current findings suggest that exercise may support B cell-mediated immunity indirectly by creating a more favorable immunological milieu; however, the functional significance of this for vaccine responses in older adults requires further investigation.

### 4.4 Exercise and the reversal of immunosenescence biomarkers

A growing body of evidence indicates that exercise can counteract the immunosenescence biomarkers—including telomere attrition, cellular senescence, SASP-driven chronic inflammation, and epigenetic signatures—and mitigate inflammaging (31, 57, 137–143). Human intervention studies have shown that engaging in regular moderate-intensity physical activity boosts immune function and slows immunosenescence progression (115, 144). Mechanistic evidence from human observational studies, reviews, and preclinical models suggests that these benefits involve restoring senescent immune cell function via autophagy (Section 5.4), mitochondrial optimization, and epigenetic reprogramming (145–147); improving systemic immune milieu by alleviating systemic inflammation (148); and reducing inflammation by modulating cytokine networks and decreasing senescent cell burden (149–151).

Epigenetic reprogramming has drawn increasing attention in human studies. Acute exercise induces histone modifications in NK cells, notably H4K5 acetylation, associated with upregulation of the activating receptor NKG2D (96). Regular exercise alters DNA methylation in promoter regions of inflammation-related genes; for instance, 6 months of high-intensity interval walking significantly increases promoter methylation of ASC, a gene involved in IL-1β and IL-18 processing (152). Moreover, older adults who engage in long-term regular exercise exhibit distinct circulating exosomal microRNA profiles (e.g., miR-486-5p, miR-215-5p) that target core metabolic pathways such as IGF-1 and correlate with improved insulin sensitivity (153). However, these findings stem largely from cross-sectional comparisons or small-sample short-term studies, and whether peripheral blood epigenetic modifications reflect changes in tissue-resident immune cells or translate into functional immune improvements remains unclear. Thus, although promising, the epigenetic evidence remains largely correlational.

Epidemiological evidence corroborates these mechanisms, showing a dose-response relationship and cumulative benefits of lifelong exercise (154–157). Thus, regular physical activity represents a promising physiological strategy that may counteract immunosenescence at cellular, epigenetic, and systemic levels.

**Figure 3:**
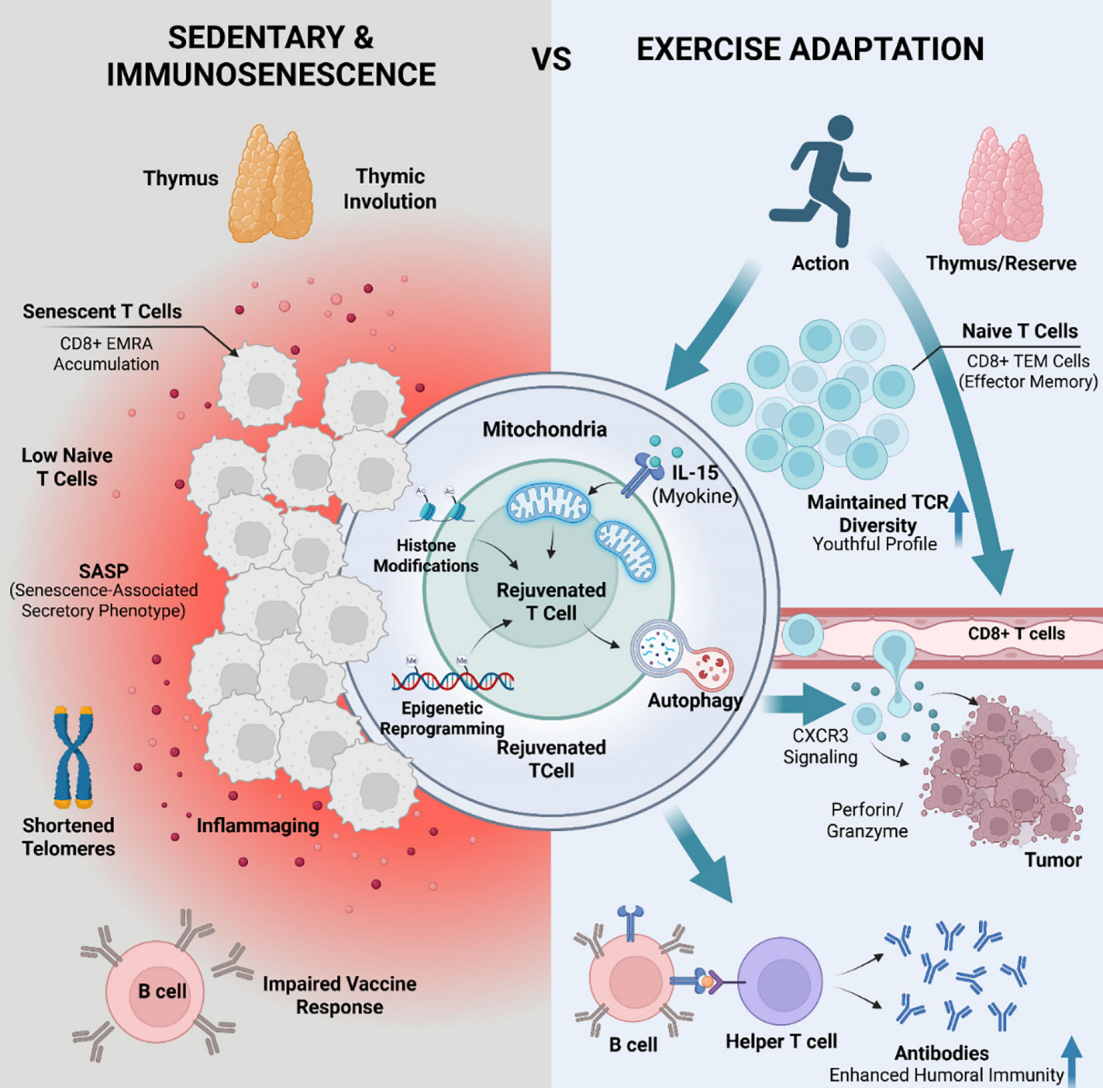
Exercise reverses key features of immunosenescence through integrated cellular and molecular mechanisms. Arrows indicate directional process flow and observed associations between biological events. Upward arrows (↑) denote increased expression or activity; downward arrows (↓) denote decreased expression or activity. This side-by-side schematic contrasts the immune phenotype of sedentary aging with exercise-induced adaptive remodeling. Sedentary lifestyle drives thymic involution, accumulation of senescent CD8^+^ EMRA T cells, shortened telomeres and SASP-mediated inflammaging (31, 57, 137–143), resulting in impaired vaccine responses (36, 67–69). Exercise preserves thymic reserve and maintains naive T cell pools and youthful TCR diversity (120, 121). Long-term moderate aerobic training increases CD8^+^ effector memory (TEM) cells while reducing senescent CD8^+^ EMRA T cells (114–119). Exercise promotes CD8^+^ T cell infiltration and function through CXCR3 signaling (88). At the cellular level, exercise rejuvenates T cells through mitochondrial repair driven by IL-15 signaling (123–125), autophagy activation clearing dysfunctional mitochondria (158, 159), and epigenetic reprogramming via histone modifications—acute exercise induces H4K5 acetylation and NKG2D upregulation (96), while regular exercise alters DNA methylation at promoter regions of inflammation-related genes such as ASC (152), and long-term exercising older adults exhibit distinct circulating exosomal microRNA profiles (153). Exercise may also modulate B cell activity (136). Created in BioRender. https://BioRender.com/u24dk8m (License: QC29THABFX).

The principal mechanisms through which exercise modulates adaptive immune function, encompassing effects on T cell subsets, T cell effector function, B cell responses, and immunosenescence biomarkers, are summarized (**Figure 3**).

## 5 Molecular and cellular mechanisms of exercise-induced immune regulation

**Figure 4:**
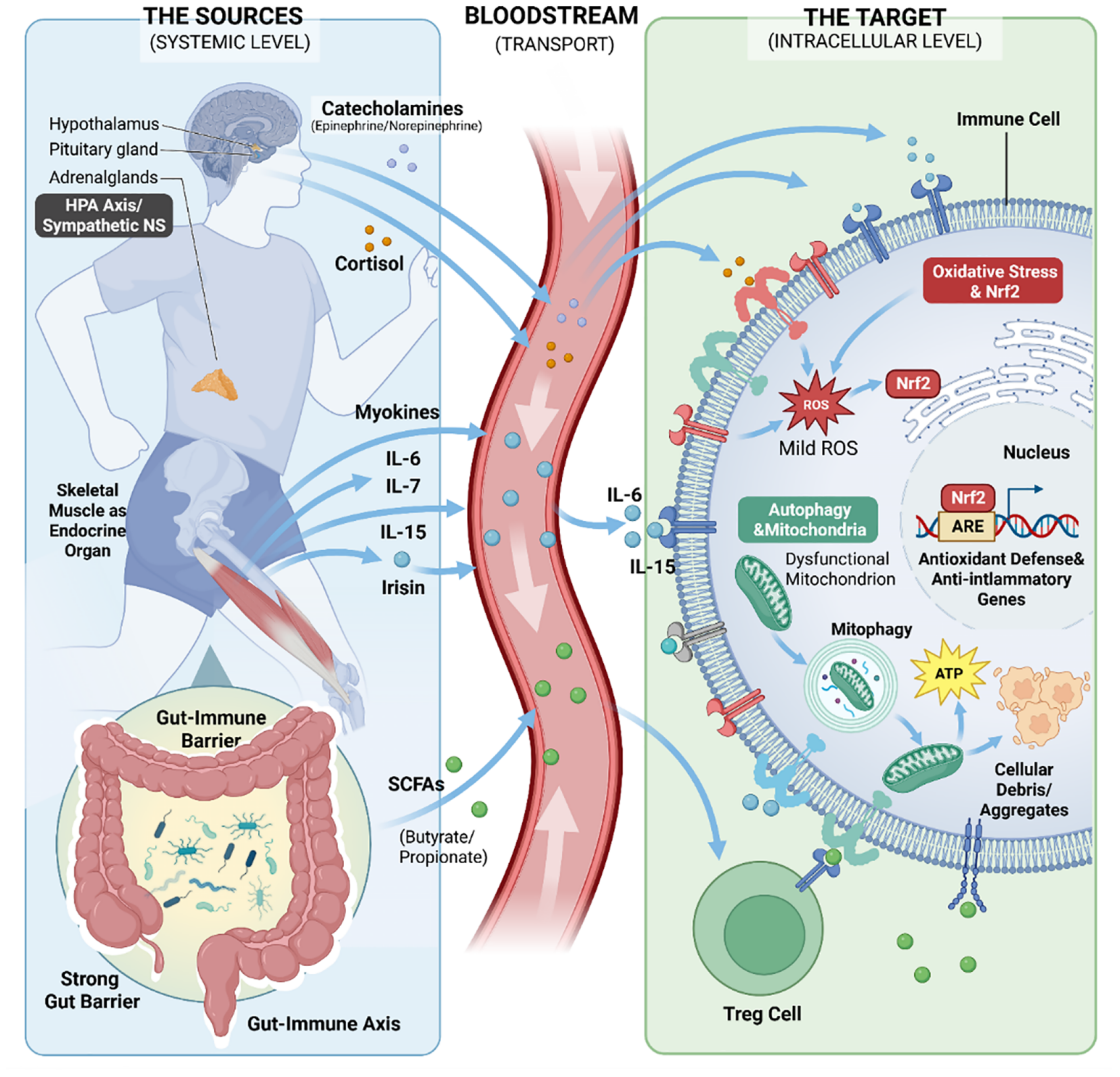
Integrative regulation signaling pathways from systemic sources to intracellular targets in exercise-induced immune. Arrows indicate directional process flow and observed associations between biological events. This schematic depicts three hierarchical levels of exercise-mediated immune regulation: systemic sources of bioactive molecules, bloodstream transport, and intracellular signaling targets. Exercise activates the hypothalamic-pituitary-adrenal axis and sympathetic nervous system, rapidly elevating plasma catecholamines (epinephrine, norepinephrine) that mobilize NK cells and neutrophils into circulation (160–162), with cortisol exerting anti-inflammatory effects during recovery (132, 163–165). Long-term training induces adaptive changes in the HPA axis, reducing basal cortisol and attenuating stress response (166). Sex hormones shape long-term immune development (167–169). Skeletal muscle acts as an endocrine organ, secreting myokines that modulate immunity (170, 171): IL-6 exerts anti-inflammatory effects by stimulating IL-10 and inhibiting TNF-α (73–75); IL-7 maintains T cell homeostasis while IL-15 promotes NK cell development and CD8^+^ T cell function (76–80); and irisin exhibits anti-inflammatory and metabolic regulatory properties (172–174). Exercise enhances gut barrier function to increase circulating SCFAs (81, 82), which promote differentiation and activity of regulatory T cells (175–177). These systemic factors collectively modulate immune cell function via Nrf2 pathway activation—exercise-induced ROS promote Nrf2 translocation from cytoplasm to nucleus, initiating transcription of ARE-driven antioxidant and anti-inflammatory genes (178–180)—and autophagy/mitophagy-mediated mitochondrial quality control that clears dysfunctional mitochondria and optimizes cellular energetics (158, 181–183).Created in BioRender. https://BioRender.com/u24dk8m (License: HC29THA3EK).

Exercise regulates immune function through multiple molecular and cellular mechanisms, including hormonal and neuroendocrine pathways, myokine signaling, oxidative stress adaptation, autophagy and mitochondrial optimization, and gut microbiota modulation. These mechanisms underpin how exercise counteracts immunosenescence (**Figure 4**).

### 5.1 Hormonal and neuroendocrine pathways

Exercise modulates immune function through neuroendocrine activation, with distinct acute and chronic components. Acute exercise rapidly elevates plasma catecholamines (epinephrine, norepinephrine), mobilizing NK cells and neutrophils into circulation and enhancing immune surveillance (160–162). Neuropeptide release may further fine-tune this acute response (184, 185). During recovery, elevated cortisol exerts anti-inflammatory and immunosuppressive effects, controlling excessive inflammation and restoring immune homeostasis (132, 163–165). Long-term exercise training induces adaptive changes in the hypothalamic–pituitary–adrenal (HPA) axis, manifested as reduced basal circulating cortisol levels and a markedly attenuated stress response to submaximal exercise stimuli; this neuroendocrine adaptation acts synergistically with the anti-inflammatory effects of exercise itself to maintain a low basal inflammatory tone (166). Sex hormones (e.g., testosterone, estrogen) shape long-term immune development and provide a foundation for adaptive changes induced by chronic training (167–169). Thus, exercise-induced hormonal changes are believed to connect physiological stress to immune adaptation, although the precise translation of these acute hormonal fluctuations into long-term immunological benefits has not been fully established, particularly in older adults. These hormonal signals are complemented by factors released directly from contracting muscles.

### 5.2 Myokines and immune regulation

Contracting skeletal muscles act as an endocrine organ during exercise, secreting myokines that modulate immunity (170, 171), while long-term training modulates basal myokine profiles and receptor sensitivity. Specifically, regular exercise is associated with a reduction in resting levels of pro-inflammatory myokines such as IL-6, while maintaining or even increasing basal levels of other immunoprotective myokines like IL-15, thereby contributing to a sustained anti-inflammatory milieu (186) A combination of mechanistic studies and reviews indicates that IL-6, which is released abundantly during exercise, exerts anti-inflammatory effects by stimulating IL-10 and inhibiting TNF-α (73–75). Preclinical studies in animal models and *in vitro* experiments have shown that other cytokines important for immune regulation also play key roles: IL-7 maintains T cell homeostasis, while IL-15, a recognized myokine, promotes NK cells development and CD8^+^ T cell function (76–80). Cellular and animal studies have demonstrated that irisin and other recently identified myokines also exhibit anti-inflammatory and metabolic regulatory properties (172–174). Thus, exercise-induced myokine release is thought to provide molecular messengers linking muscle contraction to distal immune regulation, although the causal role of individual myokines in specific immune outcomes remains to be fully established.

### 5.3 Oxidative stress and antioxidant defense

Exercise-induced oxidative stress adaptation is a cumulative process: each acute bout generates transient ROS that contribute to long-term resilience. Research in general adult populations has established that during exercise, increased metabolic demand transiently elevates reactive oxygen species (ROS) production, inducing a controlled, signaling-level oxidative stress (187–189). Similar mechanisms are presumed to operate in older adults, although age-related changes in redox balance may modify the response. This moderate oxidative stress activates intracellular adaptive defense mechanisms, primarily through the nuclear factor erythroid 2-related factor 2 (Nrf2) pathway (190). Mechanistic studies, including animal models, have shown that exercise-induced ROS promote Nrf2 translocation from cytoplasm to nucleus, where it initiates transcription of antioxidant response element (ARE)-driven genes, enhancing resistance to subsequent oxidative damage (178–180). Preclinical studies in animal models further indicate that repeated activation of this pathway through regular exercise strengthens overall antioxidant defenses, helping to mitigate chronic low-grade inflammation associated with declining immune function (180, 191, 192). Thus, exercise-induced adaptive oxidative stress provides a molecular foundation for enhancing immune system resilience and anti-inflammatory capacity. The cellular stress that activates these antioxidant pathways also serves as a potent stimulus for autophagy.

### 5.4 Autophagy and mitochondrial function

Exercise stimulates autophagy acutely after each bout, and repeated activation over time contributes to cellular quality control. Autophagy is essential for maintaining cellular homeostasis, and exercise serves as a physiological stimulus for its activation (193–197). Evidence from human, animal, and mechanistic studies indicates that two mechanisms are essential for the function of exercise-induced autophagy in immune cells. First, it clears dysfunctional mitochondria via mitophagy, optimizing cellular energetics and providing efficient ATP support for immune responses (158, 181–183). Second, autophagy facilitates removal of accumulated senescent-associated components and cellular debris (159). Senescent cell accumulation drives both immunosenescence and chronic inflammation (57, 198–201). A conceptual framework supported by reviews and preclinical studies suggests that by enhancing autophagic activity, exercise promotes turnover of aged or functionally impaired immune cells, improving the immune milieu and responsiveness (112, 145, 202). In humans, acute exercise increases autophagy markers—such as the LC3-II/LC3-I ratio and LC3 expression—in peripheral blood mononuclear cells (PBMCs) and skeletal muscle (20, 203, 204). However, interpreting these findings in the context of immunosenescence requires caution. Most human evidence relies on static measurements of autophagy-related proteins in circulating cells, which do not capture dynamic autophagic flux and may not reflect activity in tissue-resident immune cells (205). Moreover, whether exercise-induced autophagy is sufficient to clear senescent cells that accumulate with aging, and whether this translates into measurable improvements in immune function, remains an open question. Direct confirmation in older populations is needed. Thus, exercise may contribute to immune cell quality control and the clearance of damaged cellular components through autophagy, although direct evidence that exercise-induced autophagy is sufficient to reverse immunosenescence in older adults remains lacking.

### 5.5 The gut microbiota-mediated pathway

Unlike the acute neuroendocrine and myokine responses discussed above, exercise-induced gut microbiota remodeling is a chronic adaptive process. By affecting immune cell activity, the gut microbiota plays an indispensable role in the host’s immune system (206). Consistent physical activity can change the diversity and composition of the gut microbiome, such as by boosting the presence of bacterial groups that generate short-chain fatty acids (SCFAs) (81, 82). Evidence from human, animal, and mechanistic studies indicates that these microbially derived SCFAs (e.g., butyrate, propionate) exert key immunomodulatory functions. They enhance intestinal barrier integrity and reduce endotoxin translocation, thereby lowering systemic inflammation (207, 208). Moreover, studies in animal models and reviews have shown that SCFAs appear to directly affect peripheral immune cells by promoting the differentiation and activity of anti-inflammatory regulatory T cells (Tregs) to maintain immune tolerance (175–177). Thus, exercise may promote a gut-to-body immune regulatory axis by enhancing the gut microbiome and its production of beneficial metabolites, though this axis requires further validation in human interventional studies.

## 6 Optimization of exercise prescription and future directions

**Figure 5:**
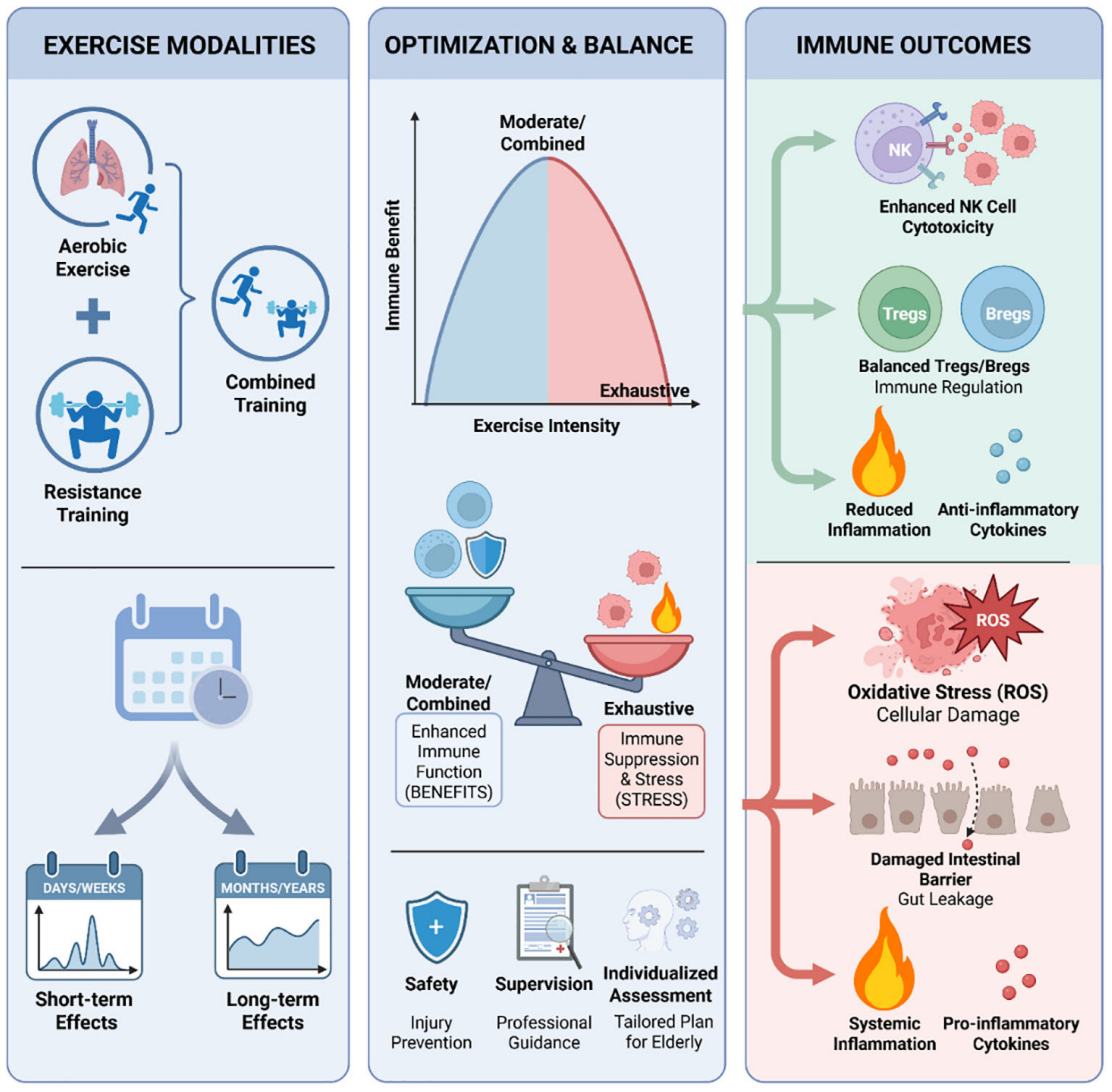
Optimizing exercise for immune enhancement: modalities, intensity, and individualization. This schematic presents a multidimensional framework for optimizing exercise prescription for immune enhancement. The left panel illustrates exercise modalities: aerobic exercise, resistance training, and combined training. A 20-week moderate-to-high-intensity combined program altered peripheral Treg and Breg proportions in elderly rheumatoid arthritis patients (209); high-intensity interval plus resistance training boosted NK cell cytotoxicity in older adults with chronic lymphocytic leukemia (210); and combined training improved CD4^+^ counts in individuals with HIV/AIDS (211). The central panel depicts the dose-response relationship: exercise intensity critically shapes immune responses via an inverted U-shaped curve. Hill et al. demonstrated graded cortisol responses to 30-min exercise: +5.7% at 40% VO_2_max, +39.9% at 60% VO_2_max, and +83.1% at 80% VO_2_max, with HPA axis activation threshold estimated at ~60% VO_2_max (165). Nine-week moderate-intensity continuous training improved leukocyte counts whereas HIIT at equivalent load reduced them (115). Exhaustive exercise can transiently suppress immunity: sustained leukocyte mitochondrial dysfunction and elevated TNF-α persisting ≥72 hours after 3 consecutive days at 85% VO_2_max, driven by oxidative stress and barrier damage (212–215). Acute exercise >1 h at high intensity suppresses lymphocyte proliferation (SMD = −0.55) (216). Chronically, 4 weeks suffices for senescent T-cell reduction (144), 12 weeks for CD8^+^ EMRA attenuation (116) and NK cell enhancement (210), and ≥24 weeks for systemic anti-inflammatory effects (217). The right panel shows immune outcomes: enhanced NK cell cytotoxicity (93), balanced Tregs/Bregs (209), and reduced inflammation (104, 217) with moderate exercise; versus oxidative stress, intestinal barrier damage and systemic inflammation with exhaustive exercise (212–215). Created in BioRender. https://BioRender.com/u24dk8m (License: CQ29TH9XD8).

Exercise prescription for immune enhancement requires balancing immunostimulatory benefits with individual physiological tolerance, with considerations including exercise type, intensity, duration, and population-specific factors (**Figure 5**).

### 6.1 Differential impact of aerobic, resistance, and combined training on immune function

Different exercise types exert distinct effects on immune function. Aerobic and resistance training each confer specific benefits, while combined regimens demonstrate synergistic potential for improving physical fitness and modulating immune markers. In a randomized controlled trial of elderly rheumatoid arthritis patients, a 20-week moderate-to-high-intensity combined program altered peripheral Treg and Breg proportions without increasing inflammatory markers (209). A pilot study in older adults with treatment-naïve chronic lymphocytic leukemia reported that a combination of high-intensity interval and resistance training boosted muscle strength and increased NK cells cytotoxicity (210). Controlled trials in individuals with HIV/AIDS have shown improved CD4^+^ counts and antioxidant capacity with combined training (211). Collectively, these findings suggest that structured combined training is a promising strategy for populations with compromised immunity. However, the evidence remains limited to small−scale studies in specific disease groups, and whether combined training is superior to aerobic or resistance training alone for immune outcomes has not been rigorously tested in large comparative trials.

### 6.2 Dose-response relationship: intensity, duration, and population-specific responses

Exercise intensity critically shapes immune responses via a complex dose-response relationship (216, 218). Current evidence permits several quantitative specifications of this relationship. Regarding intensity thresholds, Hill et al. (165) demonstrated a graded cortisol response to 30-min exercise: +5.7% (NS) at 40% VO_2_max, +39.9% at 60% VO_2_max, and +83.1% at 80% VO_2_max, with the HPA axis activation threshold estimated at approximately 60% VO_2_max. At the immune cell level, Khammassi et al. (115) showed in healthy young men that 9-week moderate-intensity continuous training (MCT) improved leukocyte (p < 0.01) and lymphocyte counts (p < 0.05) whereas HIIT at equivalent load reduced them; whether these findings generalize to older adults requires further investigation. However, exhaustive or excessive exercise can transiently suppress immunity: Tuan et al. (212) reported sustained leukocyte mitochondrial dysfunction and elevated TNF-α/sFasL persisting ≥72 hours after 3 consecutive days at 85% VO_2_max, driven by oxidative stress, systemic inflammation, immune cell dysfunction, and stress-induced barrier damage (213–215).

Regarding duration ranges, acute exercise >1.5 h suppresses immune function for 3–24 h (214), with lymphocyte proliferative suppression quantified at SMD = −0.55 for exercise >1 h at high intensity (216). Chronically, 4 weeks suffices for senescent T-cell reduction (144), 12 weeks for CD8+ EMRA T-cell differentiation attenuation (116) and NK cell enhancement in CLL patients (210), whereas systemic anti-inflammatory effects (↓TNF-α, ↓IL-6, ↑IL-10) require ≥24 weeks (217).

Regarding population-specific responses, the highest VO_2_max tertile had 17% more naïve CD8+ and 57%/37% fewer senescent CD4+/CD8+ T-cells versus the lowest tertile after adjusting for age (120); sex differences include greater cortisol responses in adult males compared to younger males (160) and female-selective IL-6 elevation after moderate exercise (219); and anti-inflammatory effects are less readily achieved in diseased versus healthy older adults (220), though combined training can still improve CD4+ counts in HIV/AIDS patients (211) and reduce Tregs/Bregs without increasing disease activity in RA (209).

Thus, exercise prescription must balance immunostimulation with physiological tolerance, requiring precise, individualized modulation. Beyond intensity, the duration of the exercise intervention, which can be acute, short-term, or long-term, fundamentally shapes the nature of immune adaptations.

### 6.3 Comparison between long-term regular exercise and short-term exercise intervention effects

The impact of long-term regular exercise on immune function is fundamentally different from that of short-term interventions. A meta-analysis of human studies has shown that acute high-intensity exercise typically induces rapid, transient fluctuations with dualistic outcomes: it may temporarily suppress lymphocyte proliferation and trigger acute inflammation (212, 216), yet human and animal studies indicate that structured short-term HIIT can also reduce oxidative stress and enhance vaccine-specific Th1 responses (221, 222). In contrast, systematic reviews and human studies long-term regular exercise promotes sustained adaptive changes, including enhanced immune cell function, attenuated inflammaging, and improved immune surveillance (7, 217, 223–226). These adaptations may extend beyond biomarker improvements to clinical benefits; for instance, observational studies have reported an association between regular physical activity and reduced risk of community-acquired infections, though randomized trial evidence establishing causality remains limited (227). Although direct evidence for protection against specific infections like COVID-19 remains limited, review articles and animal studies have suggested that maintaining an active lifestyle counteracts sedentary behavior-induced immune dysfunction (228–231). Thus, for healthy aging and lasting immune benefits, maintaining regular exercise habits appears more effective than relying on short-term interventions. A more definitive understanding of the relative merits of these two strategies, however, requires further investigation.

### 6.4 Special considerations for the aging population: safety, adherence, and individualized protocol design

When prescribing exercise for older adults, safety, long-term adherence, and individualization are paramount. Given frequent multimorbidity, immunosenescence, and reduced physiological reserve, intervention safety is the foremost priority (41, 232–234). Prescriptions must be individual-centered, integrating comprehensive assessment of disease status, treatment context, fitness level, and personal goals (235–239). Within this framework, even HIIT can be feasible and effective—improving muscle strength and immune function—in selected older patient groups under appropriate supervision (210). Personalized professional guidance is key to enhancing adherence (240–242). Integrating exercise with clinical care may yield synergistic benefits, such as optimized vaccine responses or improved quality of life during treatment (243–245). Thus, to maximize benefits and ensure ongoing participation, exercise plans for older adults should be personalized, based on comprehensive assessments, and include safety monitoring. Despite these recommendations, the current clinical evidence base for exercise immunology in aging populations has notable limitations that must be addressed.

To provide a practical, evidence-based framework for exercise prescription in older adults, **Table 2** summarizes representative studies across major exercise modalities, populations, and immune outcomes, along with their corresponding evidence levels.

**Table 2:** Summary of exercise interventions on immune function in older adults.

Exercise modality	Intensity	Duration	Study population	Key immune outcomes	Evidence level	Reference
Aerobic training	Moderate	6 months	Healthy older adults	• ↑ CD3^+^, CD4^+^, CD8^+^ T cells • ↑ IL-10 • ↓ IL-6, TNF-α	High (RCT)	(114, 116, 217)
Combined training (aerobic + resistance)	Moderate-to-high	20 weeks	Older adults with RA	• ↓ Tregs and Bregs, no increase in inflammatory markers	High (RCT)	(209)
Combined training (aerobic + resistance)	Not quantified	20 weeks	Individuals with HIV/AIDS	• ↑ CD4^+^ T cells	Moderate (RCT)	(211)
HIIT + resistance training	High	12 weeks	Older adults with CLL	• ↑ NK cells cytotoxic activity	Moderate (Pilot study)	(210)
Long-term habitual physical activity (cross-sectional)	High physical activity level (10,500–15,000 steps/day)	Long-term	Healthy older adults	• ↑ Naïve T cells, • ↑ IL-7, IL-15	Moderate (Observational)	(121)

### 6.5 Summary and analysis of current clinical research evidence

Existing clinical studies provide preliminary evidence that supervised, structured exercise interventions, including combined training and HIIT, can improve physical function and modulate immune parameters in older adults with chronic inflammatory conditions or cancer, with acceptable feasibility and safety (154, 246, 247). However, the evidence base is limited by several factors. Small sample sizes decrease both statistical power and the ability to generalize findings (229). Research has focused predominantly on specific disease populations, with insufficient investigation in healthy older adults and other common chronic disease groups (220). Long-term follow-up data are lacking; whether exercise-induced immune improvements persist or translate into meaningful clinical endpoints such as infection rates and survival remains unclear (248). Furthermore, the concept of exercise as an “immunological adjuvant” to enhance the efficacy of chemotherapy and immunotherapy represents a promising and still investigational translational direction, but its optimal integration requires validation through prospective trials, particularly in older cancer patients (249). Future research therefore necessitates larger, longer-term studies with deeper mechanistic exploration to establish precise exercise prescription guidelines for the aging population.

## 7 Conclusion

This review has synthesized evidence that regular exercise can attenuate key features of immunosenescence. Regular moderate-intensity activity improves immune function by modulating innate/adaptive immunity, curbing chronic inflammation, and reinforcing immune surveillance (225, 250). These benefits arise from coordinated multi-system interactions: neuroendocrine activation, myokine release, autophagy, mitochondrial optimization, and gut microbiota modulation (147). This positions exercise as a biological intervention in geriatric care.

Current evidence, though supportive, is constrained by small samples, short follow-ups, and limited population diversity (220, 251–253). The causal link between habitual exercise and sustained immune enhancement awaits confirmation from larger trials.

Pronounced inter-individual variability in genetics, comorbidities, and lifestyle complicates the quest for an “optimal prescription,” rendering generalized recommendations potentially ineffective (254–257). Moreover, most efficacy data derive from controlled settings, with insufficient attention to real-world adherence in older adults—a translational gap that limits the direct applicability of lab-proven protocols to community settings (154, 220, 258–260).

Future work must leverage multi-omics and long-term trials to decipher individual response heterogeneity (261–263). Prospective stratified designs and pragmatic models embedding exercise into clinical pathways (e.g., vaccine adjuvant) are needed (264–266). Bridging discovery and delivery requires robust implementation research.

It must be emphasized that many of the mechanistic insights reviewed here derive from studies in younger adults or animal models; their extrapolation to human aging must be made with caution. Direct validation in older adults remains a priority for future research.

In sum, embedding regular exercise into geriatric health management offers a cost-effective, non-pharmacological strategy to counteract immunosenescence. With interdisciplinary collaboration and continued refinement of precision−based exercise prescriptions, physical activity has the potential to become a cornerstone of healthy aging.
